# Treatments of chronic fatigue syndrome and its debilitating comorbidities: a 12-year population-based study

**DOI:** 10.1186/s12967-022-03461-0

**Published:** 2022-06-11

**Authors:** Kam-Hang Leong, Hei-Tung Yip, Chien-Feng Kuo, Shin-Yi Tsai

**Affiliations:** 1 Department of Medicine, Mackay Medical College, New Taipei City, 252 Taiwan; 2 Department of Health Policy and Management, Johns Hopkins Bloomberg School of Public Health, Johns Hopkins University, Baltimore, Maryland, 21205 USA; 3 Management Office for Health Data, China Medical University Hospital, Taichung City, 404 Taiwan; 4 Institute of Infectious Disease, Mackay Memorial Hospital, Taipei City, 104 Taiwan; 5 Department of Nursing, Nursing and Management, MacKay Junior College of Medicine, New Taipei City, 25245 Taiwan; 6 Institute of Biomedical Sciences, Mackay Medical College, New Taipei City, 252 Taiwan; 7 Institute of Long-Term Care, Mackay Medical College, New Taipei City, 252 Taiwan; 8 Department of Department of Laboratory Medicine, Mackay Memorial Hospital, Taipei, 104 Taiwan

**Keywords:** Chronic fatigue syndrome, Epidemiology, Treatment, National health programs, Nationwide population-based study

## Abstract

**Background:**

This study aims to provide 12-year nationwide epidemiology data to investigate the epidemiology and comorbidities of and therapeutic options for chronic fatigue syndrome (CFS) by analyzing the National Health Insurance Research Database.

**Methods:**

6306 patients identified as having CFS during the 2000–2012 period and 6306 controls (with similar distributions of age and sex) were analyzed.

**Result:**

The patients with CFS were predominantly female and aged 35–64 years in Taiwan and presented a higher proportion of depression, anxiety disorder, insomnia, Crohn’s disease, ulcerative colitis, renal disease, type 2 diabetes, gout, dyslipidemia, rheumatoid arthritis, Sjogren syndrome, and herpes zoster. The use of selective serotonin receptor inhibitors (SSRIs), serotonin norepinephrine reuptake inhibitors (SNRIs), Serotonin antagonist and reuptake inhibitors (SARIs), Tricyclic antidepressants (TCAs), benzodiazepine (BZD), Norepinephrine-dopamine reuptake inhibitors (NDRIs), muscle relaxants, analgesic drugs, psychotherapies, and exercise therapies was prescribed significantly more frequently in the CFS cohort than in the control group.

**Conclusion:**

This large national study shared the mainstream therapies of CFS in Taiwan, we noticed these treatments reported effective to relieve symptoms in previous studies. Furthermore, our findings indicate that clinicians should have a heightened awareness of the comorbidities of CFS, especially in psychiatric problems.

## Introduction

Chronic fatigue syndrome (CFS), also known as myalgic encephalomyelitis, is characterized by the experience of debilitating fatigue for more than 6 months that is not improved by rest [1]. The World Health Organization classifies CFS as a neurological illness, and over the last 30 years, numerous studies have identified and verified the diagnostic criteria for CFS, which are unexplained persistent or relapsing fatigue lasting at least 6 months with the addition of the concurrent presence of four or more of the following symptoms over a 6-month period: unusual postexertion fatigue, impaired memory or concentration, unrefreshing sleep, headache, muscle pain, joint pain, sore throat, and tender cervical nodes [2].

Several studies have indicated that the following multifactorial mechanisms contribute to the onset of CFS: Epstein–Barr virus, human herpes virus 6 [3], *Helicobacter pylori*,[4] *Mycobacterium tuberculosis* infection [5], immunoinflammatory pathways [6], neuroimmune dysfunctions [7], and oxidative and nitrosative stress pathways, such as those induced by burn injury [8, 9]. It also shares some features of autoimmune disease. In addition, we previously reported that inflammatory bowel disease, herpes zoster and psoriasis are associated with an increased risk of subsequent CFS [10–12].

CFS considerably reduces patients’ quality of life and places a financial burden on the patients, their families, and health care systems [13]. The primary goals of management are to relieve symptoms and provide supportive health care to improve functional capacities. However, no pharmaceutical therapies have been licensed for CFS nor has any strong evidence been revealed on the efficacy of a single regimen. In the present study, we investigated the epidemiology and comorbidities of and therapeutic options for CFS by using Taiwan’s National Health Insurance Research Database (NHIRD). Our results can help physicians diagnose CFS early and manage the disorder effectively.

## Methods

### Data source

The data set used in this study was derived from the NHIRD, which contains details concerning the demographic characteristics, dates of admission and discharge, drug prescriptions, surgical procedures, and diagnostic codes for approximately 99% of Taiwan’s population of 23 million. The 2000 Longitudinal Health Insurance Database, which is a data subset of the NHIRD, includes all the original claims data and registration files for 1 million individuals randomly sampled from among the beneficiaries of the NHI program in 2000 in Taiwan. The diseases are defined according to the *International Classification of Diseases, Ninth Revision, Clinical Modification* (*ICD-9-CM*).

### Sample participants

Cases of CFS were identified using two outpatient records or one admission record with a diagnosis of *ICD-9-CM* code 780.71. The date of the first diagnosis of CFS was the index date. For each CFS case, we used a frequency matching method to select a participant without CFS with the same sex, age, and index date as a control. Participants aged below 18 years or with missing information on sex were excluded.

### Exposure assessment and comorbidities

For this study, we examined exposure to pharmaceutical and nonpharmaceutical treatments. In terms of exposure to pharmaceutical treatments, we included the following: selective serotonin receptor inhibitors (SSRIs) (Anatomical Therapeutic Chemical (ATC) code N06AB10, N06AB06, N06AB03, N06AB08 and N06AB05), serotonin norepinephrine reuptake inhibitors (SNRIs) (ATC code N06AX21 and N06AX16), Serotonin antagonist and reuptake inhibitors (SARIs) (ATC code N06AX05), Tricyclic antidepressants (TCAs) (ATC code N06AA09 and N06CA01), benzodiazepine (BZD) (ATC code N03AE01, N05BA06, N05BA12, N05BA01, N05BA17, N05BA22, N05CD04, N05CD05, N05CD03, N05CD09, N05CD01 and N05CD08), Norepinephrine-dopamine reuptake inhibitors (NDRIs) (ATC code N06AX12), Noradrenergic and specific serotonergic antidepressants (NaSSAs) (ATC code N06AX11), muscle relaxants (ATC code M03BX08), and analgesic drugs (including acetaminophen, nonsteroidal anti-inflammatory drugs [NSAIDs], pregabalin, and gabapentin) (ATC code M02AA, D11AX18, M01A, M01B, N03AX16, and N03AX12). With regard to nonpharmaceutical treatments, we included supportive individual psychotherapy, supportive group psychotherapy, intensive individual psychotherapy, intensive group psychotherapy, reeducative individual psychotherapy, reeducative group psychotherapy, behavior modification assessments, behavior modification planning, stretching exercise, therapeutic exercise, breathing exercises, reconditioning exercise, multiple physical examinations of sleep, brainwave examination, sleep or wakefulness and a brainwave examination for sleep disorders. We made adjustments for the potentially confounding effects of other comorbidities, including depression(ICD-9-CM code 296.2, 296.3, 926.82, 300.4, 309.0, 309.1, and 311), anxiety disorder (ICD-9-CM code 300.0–300.3, 300.5–300.9, 309.2–309.4, 309.81, and 313.0), Insomnia (ICD-9-CM code 307.41, 307.42, 780.50, and 780.52), suicide (ICD-9-CM code E950-E959), crohn's disease (ICD-9-CM code 555), ulcerative colitis (ICD-9-CM code 555–556), renal disease (ICD-9-CM code 580–589), diabetes mellitus (ICD-9-CM code 250 and A181), obesity (ICD-9-CM code 278), gout (ICD-9-CM code 274), dyslipidemia (ICD-9-CM code 272), malignancy (ICD-9-CM code 140–208), HIV (ICD-9-CM code 042–044), rheumatoid arthritis (ICD-9-CM code 714), psoriasis (ICD-9-CM code 696.x), ankylosing spondylitis (ICD-9-CM code 720.0), lymphadenopathy (ICD-9-CM code 289.1–289.3, 686, and 785.6), Hashimoto’s thyroiditis (ICD-9-CM code 245.2), Sjogren’s syndrome (ICD-9-CM code 710.2), irritable bowel syndrome (ICD-9-CM code 564.1), SLE (ICD-9-CM code 710.0), celiac disease (ICD-9-CM code 579.00, fibromyalgia (ICD-9-CM 729.1), and herpes zoster (ICD-9-CM code 053) anxiety disorders, insomnia, suicide, Crohn disease, ulcerative colitis, and renal disease, prior to the index date. These were evaluated as part of the analysis.

### Statistical analysis

The descriptive statistics of CFS and the controls were reported, including demographic characteristics, comorbid diseases, and treatments received after the index date. The chi-square test was used to compare categorical variables, and Student’s t-test was used to compare continuous variables between the CFS cohort and the control cohort, as necessary. We used a logistic regression model to assess the CFS treatments the patients had received. The odds ratio (OR) and 95% confidence intervals (CIs) were calculated and then subsequently adjusted using covariates, which included age, sex, and comorbidities. Analyses were performed using SAS software (version 9.4 for Windows; SAS Institute, Cary, NC, USA). Values were considered statistically significant at p < 0.05.

## Results

**Fig. 1:**
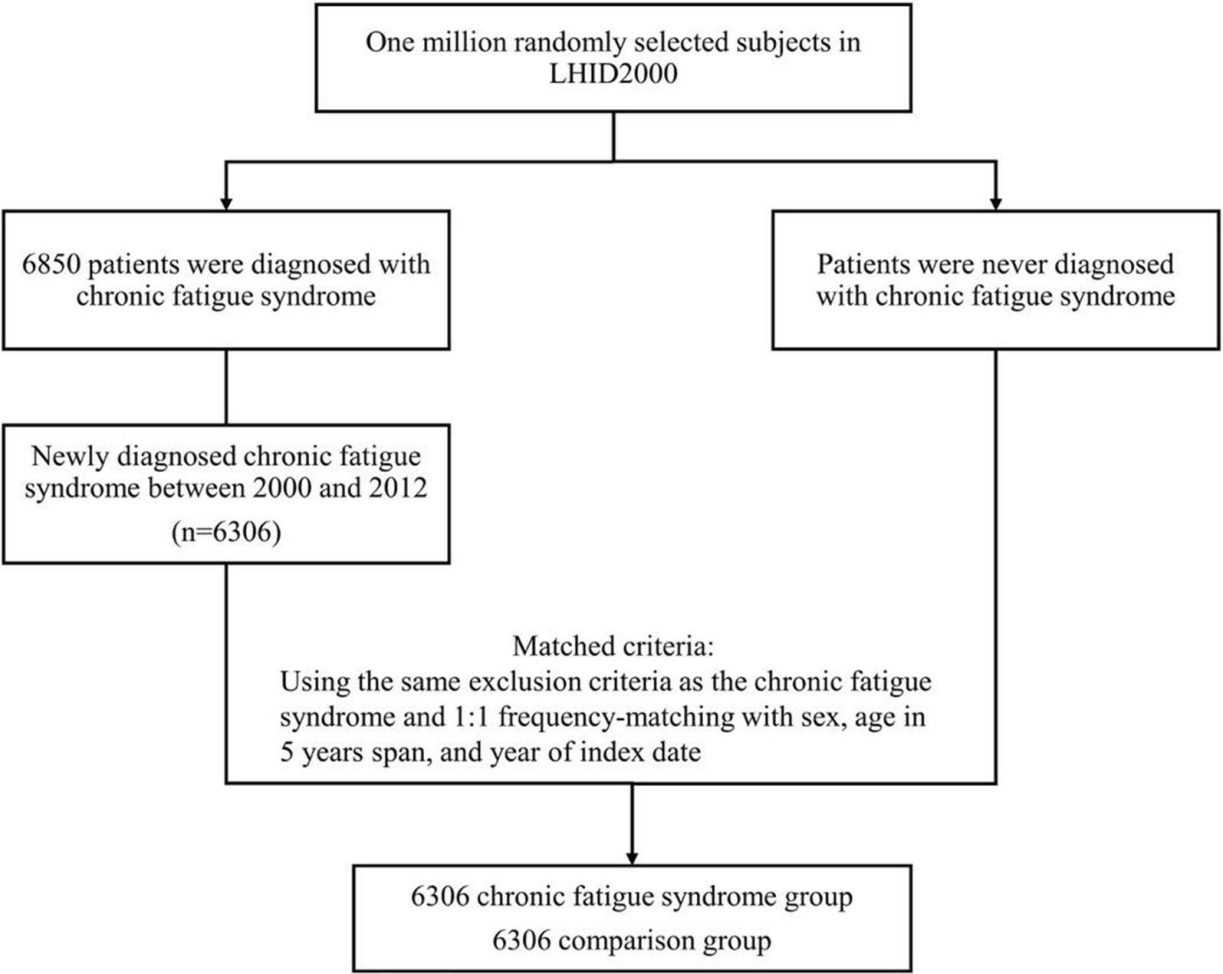
The selection process of the participants in the cohort study

Of the 1,000,000 patients in the LHID2000 database, 6850 patients were diagnosed with CFS. Among these patients, 6306 patients were newly diagnosed with CFS during the study period. In total, 12,612 participants were enrolled, including 6306 CFS patients and 6306 non-CFS patients (Fig. 1). The demographic and clinical characteristics of the study participants are presented in Table 1. The participants were predominantly female and aged 35–64 years. The mean (standard deviation) age was 50.6 years in both groups. Patients in the CFS group most presented with the comorbidities of depression, anxiety disorder, insomnia, Crohn’s disease, ulcerative colitis, renal disease, type 2 diabetes, gout, dyslipidemia, rheumatoid arthritis, Sjogren syndrome, and herpes zoster.

**Table 1:** Demographic characteristics and comorbidities of patients newly diagnosed with chronic fatigue syndrome in Taiwan between 2000 and 2012 and of those in the control group

Variable	CFS cohort	Non-CFS cohort	P-value
	(n = 6306)	(n = 6306)	
Gender			> 0.99
Female	3339 (52.9)	3339 (52.9)	
Male	2967 (47.1)	2967 (47.1)	
Age at diagnosis of CFS			> 0.99
≤ 34	1350 (21.4)	1350 (21.4)	
35–64	3485 (55.3)	3485 (55.3)	
≥ 65	1471 (23.3)	1471 (23.3)	
Age at diagnosis of CFS(mean, SD)†	50.6 (17.9)	50.6 (18.0)	0.80
Comorbidity			
Depression	807 (12.8)	407 (6.45)	< 0.0001
Anxiety disorder	2038 (32.3)	1033 (16.4)	< 0.0001
Insomnia	2303 (36.5)	1106 (17.5)	< 0.0001
Suicide	19 (0.30)	12 (0.19)	0.20
Crohn's disease	255 (4.04)	121 (1.92)	< 0.0001
Ulcerative colitis	279 (4.42)	138 (2.19)	< 0.0001
Renal disease	585 (9.28)	427 (6.77)	< 0.0001
T1DM	78 (1.24)	68 (1.08)	0.40
T2DM	1473 (23.3)	1068 (16.9)	< 0.0001
Obesity	93 (1.47)	64 (1.01)	0.01
Gout	1196 (18.9)	702 (11.1)	< 0.0001
Dyslipidemia	2252 (35.7)	1356 (21.5)	< 0.0001
Malignancy	407 (6.45)	487 (7.72)	0.01
HIV	3 (0.05)	3 (0.05)	> 0.99
Rheumatoid arthritis	254 (4.03)	155 (2.46)	< 0.0001
Psoriasis	94 (1.49)	83 (1.32)	0.40
Ankylosing spondylitis	53 (0.84)	39 (0.62)	0.14
Lymphadenopathy	132 (2.09)	104 (1.65)	0.06
Hashimoto's thyroiditis	13 (0.21)	10 (0.16)	0.53
Sjogren's syndrome	110 (1.74)	71 (1.13)	0.003
Irritable bowel syndrome	886 (14.1)	423 (6.71)	< 0.0001
Fibromyalgia	4905 (77.8)	4914 (77.9)	0.85
SLE	4 (0.06)	9 (0.14)	0.16
Herpes zoster	341 (5.41)	234 (3.71)	< 0.0001

**Fig. 2:**
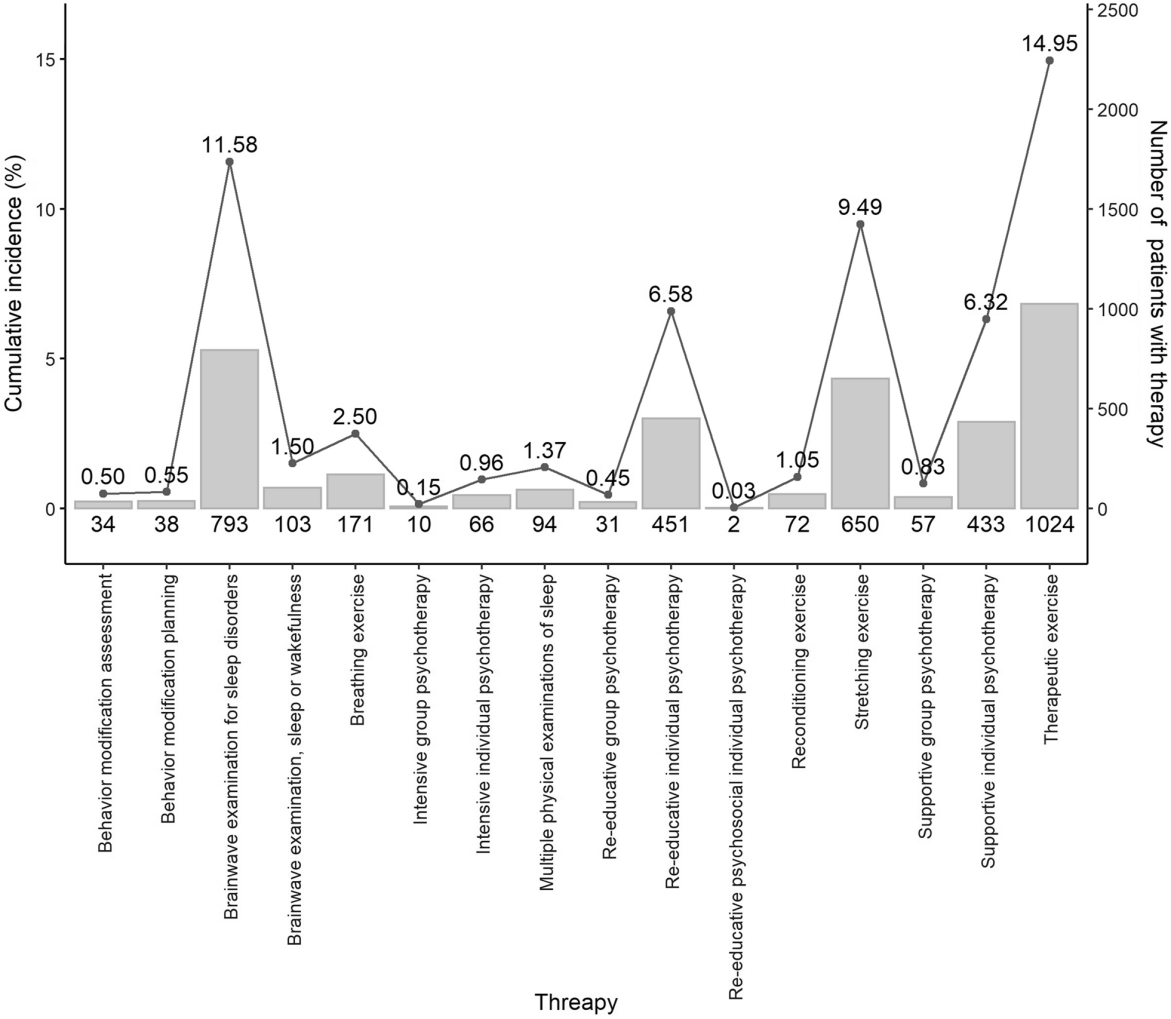
Cumulative incidences of different nonpharmaceutical treatment among the CFS subpopulations

Table 2 lists the treatments received by both the patients with CFS and those without. With adjustments for sex, age, and comorbidities, patients with CFS had higher odds of receiving SSRIs (adjusted OR [aOR] = 1.70; 95% CI 1.48, 1.95), SNRIs (adjusted OR [aOR] = 1.52; 95% CI 1.20, 1.93), SARIs (aOR = 1.56; 95% CI 1.35, 1.78), TCAs (aOR = 1.37; 95% CI 1.07, 1.76), BZD (aOR = 1.70; 95% CI 1.57, 1.84), NDRI (aOR = 1.59; 95% CI 1.08, 2.36), Muscle relaxant (aOR = 1.52; 95% CI 1.24, 1.86) and Analgesic drug (aOR = 9.55; 95% CI 7.72, 11.81) than patients without CFS. Moreover, psychotherapy, including supportive individual psychotherapy (aOR = 1.28; 95% CI 1.09, 1.51), intensive individual psychotherapy (aOR = 2.73; 95% CI 1.47, 5.04), reeducative individual psychotherapy (aOR = 1.31; 95% CI 1.11, 1.56), stretching exercises (aOR = 1.26; 95% CI 1.10, 1.45), therapeutic exercise (aOR = 1.33; 95% CI 1.19, 1.47), and a brainwave examination for sleep disorders (20001C, 20002C; aOR = 1.40; 95% CI 1.25, 1.55) were frequently prescribed to patients with CFS. Figure 2 demonstrated the cumulative incidence calculated as the number of new patients who received nonpharmaceutical treatment divided by the total number of CFS patients who were at risk and multiple by 100. In 6850 CFS patients, the highest cumulative incidences of treatment were therapeutic exercise (14.95%), followed by brainwave examination for sleep disorders (11.58%) and stretching exercise (9.49%).

**Table 2:** Odds ratios for various treatments for patients with and without chronic fatigue syndrome

Variable	N	Control		CFS		Odds ratio			
		n	%	n	%	Crude (95% CI)	p-value	Adjusted (95% CI)	p-value
SSRI						2.33 (2.05,2.66)***	< 0.001	1.70 (1.48,1.95)***	< 0.001
No	11,471	5946	52	5525	48				
Yes	1141	360	32	781	68				
SNRI						2.22 (1.77,2.78)***	< 0.001	1.52 (1.20,1.93)***	< 0.001
No	12,260	6195	51	6065	49				
Yes	352	111	32	241	68				
SARI						2.21 (1.95,2.52)***	< 0.001	1.56 (1.35,1.78)***	< 0.001
No	11,451	5927	52	5524	48				
Yes	1161	379	33	782	67				
TCAs						1.79 (1.42,2.28)***	< 0.001	1.37 (1.07,1.76)*	0.01
No	12,310	6197	50	6113	50				
Yes	302	109	36	193	64				
BZD						2.13 (1.98,2.29)***	< 0.001	1.70 (1.57,1.84)***	< 0.001
No	5368	3260	61	2108	39				
Yes	7244	3046	42	4198	58				
NDRI						2.42 (1.67,3.51)***	< 0.001	1.59 (1.08,2.36)*	0.02
No	12,476	6266	50	6210	50				
Yes	136	40	29	96	71				
NaSSA						2.02 (1.57,2.59)***	< 0.001	1.28 (0.98,1.67)	0.08
No	12,331	6212	50	6119	50				
Yes	281	94	33	187	67				
Muscle relaxant						1.80 (1.49,2.19)***	< 0.001	1.52 (1.24,1.86)***	< 0.001
No	12,150	6139	51	6011	49				
Yes	462	167	36	295	64				
Analgesic drug						12.44 (10.12,15.3)***	< 0.001	9.55 (7.72,11.81)***	< 0.001
No	1173	1071	91	102	9				
Yes	11,439	5235	46	6204	54				
Supportive individual psychotherapy						1.90 (1.63,2.21)***	< 0.001	1.28 (1.09,1.51)**	0.003
No	11,837	6032	51	5805	49				
Yes	775	274	35	501	65				
Supportive group psychotherapy						2.29 (1.52,3.45)***	< 0.001	1.52 (0.99,2.35)	0.057
No	12,504	6273	50	6231	50				
Yes	108	33	31	75	69				
Intensive individual psychotherapy						3.95 (2.20,7.12)***	< 0.001	2.73 (1.47,5.04)**	0.001
No	12,543	6292	50	6251	50				
Yes	69	14	20	55	80				
Intensive group psychotherapy						2.13 (0.92,4.93)	0.078	1.57 (0.65,3.78)	0.318
No	12,587	6298	50	6289	50				
Yes	25	8	32	17	68				
Re-educative individual psychotherapy						2.01 (1.72,2.36)***	< 0.001	1.31 (1.11,1.56)**	0.002
No	11,881	6057	51	5824	49				
Yes	731	249	34	482	66				
Re-educative group psychotherapy						2.30 (1.37,3.84)**	0.002	1.49 (0.87,2.56)	0.149
No	12,543	6285	50	6258	50				
Yes	69	21	30	48	70				
Behavior modification assessment									
No	12,612	6306	50	6306	50				
Yes	0	0	0	0	0				
Behavior modification planning						1.60 (1.02,2.54)*	0.043	1.15 (0.71,1.86)	0.582
No	12,534	6276	50	6258	50				
Yes	78	30	38	48	62				
Stretching exercise						1.44 (1.26,1.64)***	< 0.001	1.26 (1.10,1.45)***	< 0.001
No	11,600	5884	51	5716	49				
Yes	1012	422	42	590	58				
Therapeutic exercise						1.47 (1.33,1.63)***	< 0.001	1.33 (1.19,1.47)***	< 0.001
No	10,768	5535	51	5233	49				
Yes	1844	771	42	1073	58				
Breathing exercise						1.04 (0.82,1.32)	0.758	0.92 (0.71,1.18)	0.506
No	12,343	6174	50	6169	50				
Yes	269	132	49	137	51				
Reconditioning exercise						1.30 (0.94,1.79)	0.108	1.19 (0.85,1.67)	0.310
No	12,456	6238	50	6218	50				
Yes	156	68	44	88	56				
Multiple physical examinations of sleep						1.48 (1.10,2.00)*	0.011	1.07 (0.78,1.47)	0.676
No	12,434	6234	50	6200	50				
Yes	178	72	40	106	60				
Brainwave examination, sleep or wakefulness						1.60 (1.44,1.77)***	< 0.001	1.40 (1.25,1.55)***	< 0.001
No	10,825	5590	52	5235	48				
Yes	1787	716	40	1071	60				
Brainwave examination for sleep disorders									
No	12,612	6306	50	6306	50				
Yes	0	0	0	0	0				

CFS: chronic fatigue syndrome; N: total number of subjects the subgroups; n: number of subjects; CI: confidence interval; SSRI: selective serotonin receptor inhibitors; SNRI: serotonin norepinephrine Reuptake Inhibitors; SARI: serotonin antagonist and reuptake inhibitors; TCAs: tricyclic antidepressant; MAOi: Monoamine oxidase inhibitors; BZD: benzodiazepine; *P < .05, **P < .01, ***P < .001

The stratification of treatments for patients with CFS in terms of depression, anxiety disorders, and insomnia is presented in Table 3. For patients with depression, those with CFS were more likely to receive SSRIs, SNRIs, SARIs, BZD, analgesic drugs, reeducative individual psychotherapy and therapeutic exercise. SSRIs, SNRIs, SARIs, BZD, NDRI, analgesic drugs, muscle relaxants, reeducative individual psychotherapy stretching exercise and therapeutic exercise were commonly prescribed for patients with CFS identified with an anxiety disorder. In the subgroup of patients with insomnia, SSRIs, SNRIs, SARIs, BZD, analgesic drugs, reeducative individual psychotherapy and therapeutic exercise were most prescribed to patients with CFS.

**Table 3:** The odd ratios of treatments for patients with and without chronic fatigue syndrome in difference subgroup of comorbidities

Variable	Control (n = 6306)		CFS (n = 6306)		Odds ratio			
					Crude (95% CI)	p-value	Adjusted (95% CI)	p-value
	Depression							
	No	Yes	No	Yes				
SSRI					1.69 (1.29,2.22)***	< 0.001	1.52 (1.14,2.03)**	0.004
No	5633	313	4990	535				
Yes	266	94	509	272				
SNRI					2.72 (1.67,4.42)***	< 0.001	2.56 (1.55,4.23)***	< 0.001
No	5809	386	5362	703				
Yes	90	21	137	104				
SARI					1.91 (1.43,2.54)***	< 0.001	1.73 (1.28,2.36)***	< 0.001
No	5599	328	4971	553				
Yes	300	79	528	254				
TCAs					1.30 (0.78,2.17)	0.305	1.21 (0.71,2.08)	0.480
No	5812	385	5362	751				
Yes	87	22	137	56				
BZD					1.96 (1.42,2.71)***	< 0.001	1.77 (1.24,2.52)**	0.002
No	3178	82	2016	92				
Yes	2721	325	3483	715				
NDRI					1.75 (0.93,3.28)	0.083	1.60 (0.82,3.09)	0.167
No	5872	394	5447	763				
Yes	27	13	52	44				
Muscle relaxant					1.58 (0.92,2.73)	0.100	1.27 (0.72,2.25)	0.411
No	5750	389	5259	752				
Yes	149	18	240	55				
Analgesic drug					13.7 (6.41,29.15)***	< 0.001	11.15 (5.00,24.87)***	< 0.001
No	1022	49	94	8				
Yes	4877	358	5405	799				
Supportive individual psychotherapy					1.52 (1.13,2.04)**	0.006	1.27 (0.92,1.74)	0.142
No	5700	332	5204	601				
Yes	199	75	295	206				
Intensive individual psychotherapy					1.94 (0.72,5.23)	0.191	1.79 (0.58,5.46)	0.310
No	5890	402	5463	788				
Yes	9	5	36	19				
Re-educative individual psychotherapy					1.72 (1.28,2.31)***	< 0.001	1.50 (1.10,2.05)*	0.011
No	5724	333	5240	584				
Yes	175	74	259	223				
Stretching exercise					1.12 (0.77,1.64)	0.538	1.12 (0.76,1.66)	0.563
No	5522	362	5008	708				
Yes	377	45	491	99				
Therapeutic exercise					1.80 (1.30,2.50)***	< 0.001	1.81 (1.29,2.55)***	< 0.001
No	5184	351	4606	627				
Yes	715	56	893	180				
Brainwave examination, sleep or wakefulness					1.01 (0.45,2.27)	0.983	0.98 (0.42,2.30)	0.959
No	5776	398	5380	789				
Yes	123	9	119	18				

Variable	Control		CFS		Odds ratio			
					Crude (95% CI)	p-value	Adjusted (95% CI)	p-value
	Anxiety disorder							
	No	Yes	No	Yes				
SSRI					1.70 (1.38,2.09)***	< 0.001	1.54 (1.24,1.92)***	< 0.001
No	5054	892	3918	1607				
Yes	219	141	350	431				
SNRI					2.41 (1.63,3.57)***	< 0.001	2.02 (1.35,3.03)***	< 0.001
No	5194	1001	4173	1892				
Yes	79	32	95	146				
SARI					1.60 (1.31,1.96)***	< 0.001	1.37 (1.11,1.7)**	0.004
No	5045	882	3925	1599				
Yes	228	151	343	439				
TCAs					1.25 (0.86,1.83)	0.238	1.14 (0.77,1.68)	0.508
No	5204	993	4173	1940				
Yes	69	40	95	98				
BZD					1.89 (1.56,2.29)***	< 0.001	1.68 (1.37,2.06)***	< 0.001
No	3022	238	1829	279				
Yes	2251	795	2439	1759				
NDRI					2.09 (1.21,3.64)**	0.009	1.84 (1.04,3.25)*	0.037
No	5249	1017	4237	1973				
Yes	24	16	31	65				
Muscle relaxant					1.57 (1.09,2.25)*	0.015	1.46 (1.01,2.11)*	0.046
No	5147	992	4097	1914				
Yes	126	41	171	124				
Analgesic drug					7.84 (4.7,13.09)***	< 0.001	7.80 (4.55,13.38)***	< 0.001
No	1000	71	83	19				
Yes	4273	962	4185	2019				
Supportive individual psychotherapy					1.37 (1.09,1.72)**	0.007	1.15 (0.9,1.47)	0.267
No	5113	919	4063	1742				
Yes	160	114	205	296				
Intensive individual psychotherapy					2.34 (1.03,5.31)*	0.043	1.87 (0.79,4.46)	0.155
No	5266	1026	4245	2006				
Yes	7	7	23	32				
Re-educative individual psychotherapy					1.58 (1.25,2.00)***	< 0.001	1.34 (1.04,1.73)*	0.025
No	5128	929	4092	1732				
Yes	145	104	176	306				
Stretching exercise					1.49 (1.15,1.93)**	0.003	1.44 (1.1,1.88)**	0.008
No	4936	948	3918	1798				
Yes	337	85	350	240				
Therapeutic exercise					1.32 (1.08,1.61)**	0.006	1.29 (1.05,1.58)*	0.016
No	4669	866	3609	1624				
Yes	604	167	659	414				
Brainwave examination, sleep or wakefulness					0.69 (0.44,1.09)	0.109	0.67 (0.42,1.07)	0.095
No	5175	999	4178	1991				
Yes	98	34	90	47				

Variable	Control		CFS		Odds ratio			
					Crude (95% CI)	p-value	Adjusted (95% CI)	p-value
	Insomnia							
	No	Yes	No	Yes				
SSRI					1.81 (1.47,2.24)***	< 0.001	1.65 (1.32,2.06)***	< 0.001
No	4969	977	3667	1858				
Yes	231	129	336	445				
SNRI					1.74 (1.23,2.47)**	0.002	1.54 (1.07,2.21)*	0.019
No	5131	1064	3910	2155				
Yes	69	42	93	148				
SARI					1.45 (1.2,1.76)***	< 0.001	1.30 (1.06,1.59)*	0.011
No	4991	936	3701	1823				
Yes	209	170	302	480				
TCAs					1.62 (1.09,2.4)*	0.018	1.58 (1.05,2.38)*	0.027
No	5124	1073	3919	2194				
Yes	76	33	84	109				
BZD					1.43 (1.2,1.71)***	< 0.001	1.37 (1.14,1.66)**	0.001
No	3008	252	1714	394				
Yes	2192	854	2289	1909				
NDRI					2.01 (1.14,3.55)*	0.016	1.75 (0.97,3.16)	0.062
No	5175	1091	3969	2241				
Yes	25	15	34	62				
Muscle relaxant					1.19 (0.86,1.65)	0.285	1.11 (0.80,1.55)	0.530
No	5087	1052	3841	2170				
Yes	113	54	162	133				
Analgesic drug					8.75 (5.77,13.25)***	< 0.001	8.00 (5.16,12.4)***	< 0.001
No	960	111	73	29				
Yes	4240	995	3930	2274				
Supportive individual psychotherapy					1.26 (1.01,1.59)*	0.044	1.03 (0.81,1.32)	0.784
No	5042	990	3799	2006				
Yes	158	116	204	297				
Intensive individual psychotherapy					1.63 (0.74,3.6)	0.228	1.31 (0.57,3.02)	0.519
No	5194	1098	3975	2276				
Yes	6	8	28	27				
Re-educative individual psychotherapy					1.57 (1.24,2)***	< 0.001	1.35 (1.04,1.74)*	0.024
No	5049	1008	3826	1998				
Yes	151	98	177	305				
Stretching exercise					1.30 (1.02,1.66)*	0.033	1.26 (0.99,1.62)	0.064
No	4878	1006	3677	2039				
Yes	322	100	326	264				
Therapeutic exercise					1.52 (1.25,1.84)***	< 0.001	1.52 (1.25,1.86)***	< 0.001
No	4591	944	3406	1827				
Yes	609	162	597	476				
Brainwave examination, sleep or wakefulness					0.84 (0.56,1.26)	0.405	0.88 (0.58,1.33)	0.539
No	5106	1068	3933	2236				
Yes	94	38	70	67				

CFS: chronic fatigue syndrome; CI: confidence interval; *P < .05, **P < .01, ***P < .001

As presented in Table 4, patients with CFS were more likely to receive SSRIs, BZD and analgesic drugs in each age group. The odds of patients with CFS aged 35–64 and ≥ 65 receiving SARIs and muscle relaxant treatments were higher than the odds of those without CFS. For participants aged 35–64 years, reeducative individual psychotherapy was also frequently received by patients with CFS. Female and male patients with CFS were equally likely to be treated with SSRIs, SNRIs, SARIs, BZD, muscle relaxants, analgesic drugs, reeducative individual psychotherapy, intensive individual psychotherapy and therapeutic exercise, TCAs was higher prescribed to female and NDRI was higher used in male, as presented in Table 5.

**Table 4:** The odd ratios of treatments for patients with and without chronic fatigue syndrome in difference subgroup of age

Variable	Control (n = 6306)		CFS (n = 6306)		Odds ratio			
	Age ≤ 34 y/o				Crude (95% CI)	p-value	Adjusted (95% CI)	p-value
	No	Yes	No	Yes				
SSRI					1.98 (1.43,2.74)***	< 0.001	1.53 (1.08,2.15)*	0.015
No	4758	1188	4386	1139				
Yes	300	60	667	114				
SNRI					2.13 (1.23,3.7)**	0.007	1.39 (0.77,2.52)	0.272
No	4966	1229	4852	1213				
Yes	92	19	201	40				
SARI					1.82 (1.25,2.65)**	0.002	1.20 (0.8,1.8)	0.375
No	4724	1203	4351	1173				
Yes	334	45	702	80				
TCAs					2.17 (1.12,4.21)*	0.022	1.59 (0.78,3.22)	0.2
No	4962	1235	4888	1225				
Yes	96	13	165	28				
BZD					1.91 (1.62,2.25)***	< 0.001	1.61 (1.36,1.92)***	< 0.001
No	2380	880	1411	697				
Yes	2678	368	3642	556				
NDRI					2.29 (0.94,5.59)	0.068	1.34 (0.5,3.56)	0.557
No	5025	1241	4973	1237				
Yes	33	7	80	16				
Muscle relaxant					1.88 (1.1,3.21)*	0.021	1.69 (0.97,2.96)	0.065
No	4912	1227	4797	1214				
Yes	146	21	256	39				
Analgesic drug					3.94 (2.57,6.02)***	< 0.001	3.89 (2.49,6.06)***	< 0.001
No	968	103	74	28				
Yes	4090	1145	4979	1225				
Supportive individual psychotherapy					1.74 (1.23,2.45)**	0.002	1.13 (0.77,1.64)	0.531
No	4839	1193	4645	1160				
Yes	219	55	408	93				
Intensive individual psychotherapy					5.37 (1.56,18.47)**	0.008	3.34 (0.92,12.17)	0.067
No	5047	1245	5014	1237				
Yes	11	3	39	16				
Re-educative individual psychotherapy					1.85 (1.29,2.65)***	< 0.001	1.20 (0.81,1.79)	0.362
No	4858	1199	4659	1165				
Yes	200	49	394	88				
Stretching exercise					1.21 (0.86,1.70)	0.274	1.15 (0.81,1.63)	0.425
No	4701	1183	4541	1175				
Yes	357	65	512	78				
Therapeutic exercise					1.08 (0.84,1.39)	0.544	0.98 (0.76,1.28)	0.89
No	4418	1117	4121	1112				
Yes	640	131	932	141				
Brainwave examination, sleep or wakefulness					0.63 (0.24,1.64)	0.344	0.60 (0.22,1.65)	0.321
No	4937	1237	4923	1246				
Yes	121	11	130	7				

Variable	Control (n = 6306)		CFS (n = 6306)		Odds ratio			
	Age 35–64 y/o				Crude (95% CI)	p-value	Adjusted (95% CI)	p-value
	No	Yes	No	Yes				
SSRI					2.15 (1.85,2.50)***	< 0.001	1.57 (1.34,1.85)***	< 0.001
No	1389	4557	1251	4274				
Yes	82	278	220	561				
SNRI					2.23 (1.71,2.90)***	< 0.001	1.56 (1.18,2.07)**	0.002
No	1443	4752	1411	4654				
Yes	28	83	60	181				
SARI					2.19 (1.88,2.55)***	< 0.001	1.53 (1.3,1.81)***	< 0.001
No	1356	4571	1232	4292				
Yes	115	264	239	543				
TCAs					2.09 (1.57,2.79)***	< 0.001	1.66 (1.22,2.24)**	0.001
No	1432	4765	1422	4691				
Yes	39	70	49	144				
BZD					2.15 (1.98,2.33)***	< 0.001	1.71 (1.56,1.87)***	< 0.001
No	474	2786	236	1872				
Yes	997	2049	1235	2963				
NDRI					2.19 (1.46,3.3)***	< 0.001	1.52 (0.99,2.35)	0.057
No	1465	4801	1449	4761				
Yes	6	34	22	74				
Muscle relaxant					1.73 (1.38,2.18)***	< 0.001	1.46 (1.14,1.85)**	0.002
No	1424	4715	1380	4631				
Yes	47	120	91	204				
Analgesic drug					9.18 (7.27,11.59)***	< 0.001	6.83 (5.38,8.66)***	< 0.001
No	410	661	20	82				
Yes	1061	4174	1451	4753				
Supportive individual psychotherapy					1.82 (1.53,2.16)***	< 0.001	1.20 (1,1.45)	0.054
No	1415	4617	1352	4453				
Yes	56	218	119	382				
Intensive individual psychotherapy					4.11 (2.19,7.75)***	< 0.001	2.95 (1.52,5.73)**	0.001
No	1469	4823	1465	4786				
Yes	2	12	6	49				
Re-educative individual psychotherapy					2.01 (1.68,2.39)***	< 0.001	1.33 (1.1,1.61)**	0.004
No	1423	4634	1376	4448				
Yes	48	201	95	387				
Stretching exercise					1.43 (1.23,1.67)***	< 0.001	1.27 (1.08,1.49)**	0.004
No	1360	4524	1315	4401				
Yes	111	311	156	434				
Therapeutic exercise					1.42 (1.26,1.59)***	< 0.001	1.28 (1.13,1.45)***	< 0.001
No	1256	4279	1150	4083				
Yes	215	556	321	752				
Brainwave examination, sleep or wakefulness					1.08 (0.79,1.50)	0.622	0.98 (0.69,1.37)	0.889
No	1411	4763	1412	4757				
Yes	60	72	59	78				

Variable	Control (n = 6306)		CFS (n = 6306)		Odds ratio			
	Age ≥ 65 y/o				Crude (95% CI)	p-value	Adjusted (95% CI)	p-value
	No	Yes	No	Yes				
SSRI					2.98 (2.29,3.88)***	< 0.001	2.17 (1.64,2.88)***	< 0.001
No	4557	1389	4274	1251				
Yes	278	82	561	220				
SNRI					2.19 (1.39,3.45)***	< 0.001	1.46 (0.9,2.37)	0.121
No	4752	1443	4654	1411				
Yes	83	28	181	60				
SARI					2.29 (1.81,2.89)***	< 0.001	1.69 (1.31,2.17)***	< 0.001
No	4571	1356	4292	1232				
Yes	264	115	543	239				
TCAs					1.27 (0.83,1.94)	0.28	0.89 (0.56,1.42)	0.633
No	4765	1432	4691	1422				
Yes	70	39	144	49				
BZD					2.49 (2.08,2.97)***	< 0.001	1.72 (1.42,2.09)***	< 0.001
No	2786	474	1872	236				
Yes	2049	997	2963	1235				
NDRI					3.71 (1.5,9.17)**	0.005	2.33 (0.9,6.03)	0.082
No	4801	1465	4761	1449				
Yes	34	6	74	22				
Muscle relaxant					2.00 (1.39,2.86)***	< 0.001	1.75 (1.2,2.56)**	0.004
No	4715	1424	4631	1380				
Yes	120	47	204	91				
Analgesic drug					28.0 (17.77,44.22)***	< 0.001	27.1 (16.65,44.03)***	< 0.001
No	661	410	82	20				
Yes	4174	1061	4753	1451				
Supportive individual psychotherapy					2.22 (1.6,3.08)***	< 0.001	1.58 (1.11,2.24)*	0.01
No	4617	1415	4453	1352				
Yes	218	56	382	119				
Intensive individual psychotherapy					3.01 (0.61,14.93)	0.178	1.47 (0.26,8.18)	0.662
No	4823	1469	4786	1465				
Yes	12	2	49	6				
Re-educative individual psychotherapy					2.05 (1.44,2.92)***	< 0.001	1.28 (0.87,1.88)	0.207
No	4634	1423	4448	1376				
Yes	201	48	387	95				
Stretching exercise					1.45 (1.13,1.88)**	0.004	1.29 (0.99,1.69)	0.063
No	4524	1360	4401	1315				
Yes	311	111	434	156				
Therapeutic exercise					1.63 (1.35,1.97)***	< 0.001	1.48 (1.21,1.82)***	< 0.001
No	4279	1256	4083	1150				
Yes	556	215	752	321				
Brainwave examination, sleep or wakefulness					0.98 (0.68,1.42)	0.925	0.86 (0.58,1.27)	0.447
No	4763	1411	4757	1412				
Yes	72	60	78	59				

CFS: chronic fatigue syndrome; CI: confidence interval; *:p-value; *P < .05, **P < .01, ***P < .001

**Table 5:** The odd ratios of treatments for patients with and without chronic fatigue syndrome in difference subgroup of sex

Variable	Control (n = 6306)		CFS (n = 6306)		Odds ratio			
	Female				Crude (95% CI)	p-value	Adjusted (95% CI)	p-value
	No	Yes	No	Yes				
SSRI					2.36 (1.99,2.81)***	< 0.001	1.71 (1.42,2.06)***	< 0.001
No	2815	3131	2639	2886				
Yes	152	208	328	453				
SNRI					2.04 (1.51,2.75)***	< 0.001	1.42 (1.04,1.95)*	0.029
No	2922	3273	2858	3207				
Yes	45	66	109	132				
SARI					2.10 (1.77,2.5)***	< 0.001	1.46 (1.21,1.76)***	< 0.001
No	2805	3122	2611	2913				
Yes	162	217	356	426				
TCAs					2.25 (1.62,3.13)***	< 0.001	1.69 (1.2,2.38)**	0.003
No	2911	3286	2891	3222				
Yes	56	53	76	117				
BZD					2.22 (2.01,2.46)***	< 0.001	1.71 (1.53,1.92)***	< 0.001
No	1663	1597	1133	975				
Yes	1304	1742	1834	2364				
NDRI					2.16 (1.28,3.63)**	0.004	1.39 (0.8,2.4)	0.241
No	2948	3318	2916	3294				
Yes	19	21	51	45				
Muscle relaxant					1.89 (1.45,2.45)***	< 0.001	1.52 (1.15,2.01)**	0.003
No	2889	3250	2836	3175				
Yes	78	89	131	164				
Analgesic drug					13.54 (9.80,18.7)***	< 0.001	10.11 (7.26,14.09)***	< 0.001
No	590	481	61	41				
Yes	2377	2858	2906	3298				
Supportive individual psychotherapy					1.74 (1.42,2.13)***	< 0.001	1.19 (0.96,1.48)	0.121
No	2851	3181	2731	3074				
Yes	116	158	236	265				
Intensive individual psychotherapy					4.03 (1.85,8.76)***	< 0.001	2.76 (1.21,6.3)*	0.016
No	2961	3331	2944	3307				
Yes	6	8	23	32				
Re-educative individual psychotherapy					1.98 (1.61,2.44)***	< 0.001	1.26 (1.01,1.59)*	0.045
No	2860	3197	2755	3069				
Yes	107	142	212	270				
Stretching exercise					1.49 (1.26,1.77)***	< 0.001	1.30 (1.09,1.56)**	0.004
No	2787	3097	2726	2990				
Yes	180	242	241	349				
Therapeutic exercise					1.39 (1.22,1.59)***	< 0.001	1.24 (1.07,1.43)**	0.003
No	2637	2898	2477	2756				
Yes	330	441	490	583				
Brainwave examination, sleep or wakefulness					1.42 (0.99,2.04)	0.057	1.18 (0.81,1.74)	0.393
No	2886	3288	2902	3267				
Yes	81	51	65	72				

Variable	Control (n = 6306)		CFS (n = 6306)		Odds ratio			
	Male				Crude (95% CI)	p-value	Adjusted (95% CI)	p-value
	No	Yes	No	Yes				
SSRI					2.3 (1.89,2.81)***	< 0.001	1.70 (1.37,2.10)***	< 0.001
No	3131	2815	2886	2639				
Yes	208	152	453	328				
SNRI					2.48 (1.74,3.52)***	< 0.001	1.64 (1.13,2.38)**	0.009
No	3273	2922	3207	2858				
Yes	66	45	132	109				
SARI					2.36 (1.95,2.86)***	< 0.001	1.71 (1.38,2.10)***	< 0.001
No	3122	2805	2913	2611				
Yes	217	162	426	356				
TCAs					1.37 (0.96,1.94)	0.079	1.06 (0.73,1.53)	0.771
No	3286	2911	3222	2891				
Yes	53	56	117	76				
BZD					2.06 (1.86,2.29)***	< 0.001	1.69 (1.50,1.90)***	< 0.001
No	1597	1663	975	1133				
Yes	1742	1304	2364	1834				
NDRI					2.71 (1.60,4.61)***	< 0.001	1.81 (1.03,3.17)*	0.039
No	3318	2948	3294	2916				
Yes	21	19	45	51				
Muscle relaxant					1.71 (1.29,2.28)***	< 0.001	1.53 (1.13,2.07)**	0.005
No	3250	2889	3175	2836				
Yes	89	78	164	131				
Analgesic drug					11.82 (9.03,15.47)***	< 0.001	9.40 (7.11,12.43)***	< 0.001
No	481	590	41	61				
Yes	2858	2377	3298	2906				
Supportive individual psychotherapy					2.12 (1.69,2.67)***	< 0.001	1.38 (1.08,1.77)*	0.012
No	3181	2851	3074	2731				
Yes	158	116	265	236				
Intensive individual psychotherapy					3.86 (1.57,9.48)**	0.003	2.56 (1.00,6.57)	0.051
No	3331	2961	3307	2944				
Yes	8	6	32	23				
Re-educative individual psychotherapy					2.06 (1.62,2.61)***	< 0.001	1.39 (1.07,1.80)*	0.014
No	3197	2860	3069	2755				
Yes	142	107	270	212				
Stretching exercise					1.37 (1.12,1.67)**	0.002	1.21 (0.98,1.49)	0.081
No	3097	2787	2990	2726				
Yes	242	180	349	241				
Therapeutic exercise					1.58 (1.36,1.84)***	< 0.001	1.44 (1.23,1.69)***	< 0.001
No	2898	2637	2756	2477				
Yes	441	330	583	490				
Brainwave examination, sleep or wakefulness					0.8 (0.57,1.11)	0.181	0.75 (0.53,1.07)	0.117
No	3288	2886	3267	2902				
Yes	51	81	72	65				

CFS: chronic fatigue syndrome; CI: confidence interval;*P < .05, **P < .01, ***P < .001

## Discussion

Our nationwide population-based study revealed that patients with CFS experienced more comorbidities, such as psychiatric problems (depression, anxiety disorders, and insomnia), autoimmune diseases (Crohn disease, ulcerative colitis, rheumatoid arthritis, and Sjogren syndrome), type 2 diabetes, renal diseases, and malignancy, than the participants without CFS. In addition, we found that the use of SSRIs, SARIs, SNRIs, TCAs, NDRI, BZD, muscle relaxants, analgesic drugs, psychotherapies and exercise therapies were higher in the CFS cohort. This finding is consistent with the general treatment for CFS [14]. Notably, brainwave examination is not a standard examination method for diagnosing CFS, but it was regularly used by clinicians in our study.

The etiology of CFS remains unknown. Emerging research suggests CFS is an autoimmune disease, with evidence of dysregulation of the immune and autonomic nervous systems as well as metabolic disturbances, triggered particularly by infection with stress [15]. Patients with CFS have been identified as having increased levels of autoantibodies against ß2-adrenergic receptors and M3 acetylcholine receptors [16]. The hypothalamic–pituitary–adrenal (HPA) axis maintains homeostasis through a self-regulating feedback system that helps to manage stress [17, 18], and abnormalities in the HPA axis are believed to be a feature of CFS [19]. In addition, we previously reported that psoriasis and inflammatory bowel disease significantly increased the risk of CFS [10, 11]. In future studies, the aspects of CFS linked to autoimmune diseases should be clarified.

In recent 2 years during COVID-19 pandemic, many studies indicated that some COVID-19 patients had persistent clinical signs and symptoms including fatigue, breathlessness, and cognitive dysfunction after recovering from initial illness. This condition named Post COVID-19 Syndrome or long COVID. Pathological inflammation with immune dysfunction was a one of the underlying multifactorial mechanism of long COVID, which was similar to CFS [20–22]. Various autoantibodies were found in 10–50% of patients with COVID-19 [23]. These autoantibodies and increased levels of pro-inflammatory markers contributed to the disease severity and inflammation-related symptoms such as fatigue and joint pain [22, 24]. The treatments of CFS were believed to have a potential effect of relieving fatigue in long COVID cases [22, 25]. Future studies should be conducted to determine the underlying mechanism and treatments between CFS and long COVID.

Our comparison of patients with CFS with those without demonstrated that the use of SSRIs, SNRIs, SARIs and BZD was higher in the CFS cohort after adjustments for age, sex, and comorbidities (Table 2), especially in those with psychiatric problems (depression, anxiety disorders, and insomnia; Table 3). However, a subclassification analysis of age and sex established no significant differences between the two groups (Tables 4 and 5). Patients with CFS have been reported to have clinical depression and anxiety [26], and several pathophysiologies related to depression have been reported, such as inflammation with elevated cytokine levels (e.g., interleukin [IL]-1, tumor necrosis factor alpha [TNF-α]), increased oxidative stress, and decreased neurotrophic factors and brain neurotransmitters [27]. Serotonin (or 5-hydroxytryptamine 1A [5-HT1A]), a monoamine neurotransmitter, has been discovered to be linked to mood, behavior, sleep cycles, and appetite [28]. One study indicated that the number of brain 5-HT1A receptors was decreased in patients with CFS, with the decrease particularly marked in the bilateral hippocampus [29]. Furthermore, changes in the HPA axis in chronic stress were reported to be associated with the serotonin system and abnormal adrenocortical activity and were observed in patients with CFS [30]. One study indicated that patients with CFS prescribed SSRIs had a faster rate of recovery and experienced a greater reduction in fatigue levels than untreated patients [31]. However, few clinical trials have been conducted on CFS treatments, although the use of SSRIs for fibromyalgia, especially for patients with depression, may be advantageous for CFS [32]. Bupropion, a norepinephrine-dopamine reuptake inhibitor (NDRI), was reported to improve hypersomnia and fatigue significantly in the patients with major depressive disorder compared with the placebo-group [33]. Unrefreshing sleep is one feature of CFS, Cognitive-behavioral therapy for insomnia (CBT-I) and sleep hygiene education should be applied whenever possible [34]. Experienced clinicians believed that low-dose TCAs and BZD may also be useful for sleep. However, monitoring the adverse effects including drowsiness upon awakening must be considered.

Treatments for pain symptoms, including muscle relaxants and analgesic drugs, were more common among the CFS cohort (Table 2), but no significant difference in psychiatric comorbidities, age, or sex was identified in the subclassification analysis (Tables 3, 4, and 5). Chronic pain in the muscles, joints, and subcutaneous tissues was a common presenting symptom in patients with CFS. The potential contributing mechanisms may be oxidative and nitrosative stress, low-grade inflammation, and impaired heat shock protein production [35]. Another hypothesis concerning muscle fatigue is that it results from the overutilization of the lactate dehydrogenase pathway and slowed acid clearance after exercise [36]. The mainstream management of pain in CFS is similar to that for fibromyalgia. Pain can be treated with NSAIDs or acetaminophen. Pregabalin or gabapentin are helpful for neuropathic and fibromyalgia pain [37]; however, clinicians should be aware of the adverse effects of this treatment on cognitive dysfunction and weight gain. One systematic review indicated that cyclobenzaprine was more effective for back pain [38] but was associated with the side effects of drowsiness, dizziness, and dry mouth. Nonpharmacologic interventions for pain vary, and useful modalities include meditation, warm baths, massage, stretching, acupuncture, hydrotherapy, chiropractic, yoga, tai chi, and transcutaneous electrical nerve stimulation [14, 39].

According to the information released by NHIRD and clinical experiences, the supportive individual psychotherapy is performed by various professional members in psychiatric team under the psychiatrists’ guidance. The re-educative individual psychotherapy is mainly performed by psychotherapists and the intensive individual psychotherapy is administered by psychiatrists. Our results found the application of all psychotherapy was higher in the CFS cohort since those with psychiatric problems are mostly referred to psychotherapists for re-educative individual psychotherapy. However, the group psychotherapy is not a first choice for clinicians in Taiwan. In the age and sex subclassification analysis, psychotherapy was not prescribed significantly more frequently to young aged (below or equal to 34 y/o) patients. With regard to nonpharmaceutical options, cognitive behavioral therapy (CBT), a psychotherapy, has been prescribed to patients with CFS. CBT includes relaxation exercises, the development of coping mechanisms, and stress management, and it is an effective treatment for depression and anxiety and eating and panic disorders [40]. One randomized trial reported that CBT and graded exercise therapy (GET) were safe for CFS and effective at improving fatigue and functional impairment [41, 42]. A 16 week standard individual CBT has been shown to be beneficial in physical function and fatigue [43]. Furthermore, CBT is the most cost-effective treatment option for CFS [44]. Although CBT is often used with GET, the program should be discussed with patients to ensure their compliance.

Brainwave examination was also significantly more frequently prescribed in the CFS cohort (OR = 1.40; Table 2), regardless of whether the participant had depression, an anxiety disorder, or insomnia (Table 3). On the other hand, polysomnography (PSG), including brainwave examination (EEG), eye movements (EOG), muscle activity or skeletal muscle activation (EMG), and heart rhythm (ECG) records certain body functions during sleeping, Nonrestorative sleep is a key feature of CFS and is defined as the subjective experience that sleep has not been sufficiently refreshing or restorative [45, 46], resulting in increased daytime drowsiness, mental fatigue, and neurocognitive impairment [47]. Primary sleep disorders (PSDs), including primary insomnia, obstructive sleep apnea, periodic limb movement disorder, and narcolepsy, occur in approximately 18% of patients with CFS [48]. PSG is a key tool for detecting these disorders. Patients with more severe symptoms should be routinely screened for PSDs with appropriate questionnaires, a semistructured history interview, and PSG [49].

Some emerging management strategies for CFS have been proposed in recent years. The fact that drugs targeting immune responses or impaired autoregulation of blood flow was indicated to be effectual in CFS [50]. We previously discovered that the increased risk of CFS among patients with psoriasis was attenuated by immunomodulatory drugs [11]. In addition, a small placebo-controlled and open study mentioned that rituximab achieved sustained clinical responses in patients with CFS [51], and a clinical trial demonstrated that rintatolimod, a restricted toll-like receptor 3 agonist, achieved significant improvements in patients with CFS [52]. Furthermore, increased levels of several cytokines, including IL-1 and TNF-α, have been positively correlated with fatigue [53]. These findings provide insight into treating CFS through immune pathways. Another emerging treatment of CFS is dietary intervention, with one systemic review indicating that nicotinamide adenine dinucleotide hydride, coenzyme Q10, and probiotic supplements relieved CFS symptoms [54]. These potential mechanisms contribute by increasing adenosine triphosphate production and improving gut microbiota. Aripiprazole was reported to relieve the symptoms of CFS including fatigue and unrefreshing sleep effectively [55]. Biofeedback therapy has also demonstrated benefits in the treatment of CFS. Compared with GET, heart rate variability biofeedback therapy has improved quality of life in cases of mental health disorders, including depression, potentially through the enhancement of self-efficacy and self-control [56].

Our study has some limitations. First, the severity of CFS and efficacy of the treatment were not evaluated in the study because of limited information available in the NHIRD. Second, some nonpharmaceutical treatments, such as meditation and massage, were not included in our study because they were not included in the database. Third, patients’ personal information and family histories, such as symptoms, occupation, and laboratory data, were not available because of the anonymity of the NHIRD. Fourth, incorrect coding and diagnoses in the database may have resulted in bias in the data analysis; however, such errors may result in considerable penalties for physicians, and hence, they are unlikely. Moreover, data on 99.9% of Taiwan’s population are contained in the NHIRD, making the database a robust source of data, the reliability and validity of which have been reported previously [57]. Consequently, the diagnoses and codes should be reliable in our study.

## Conclusion

In our nationwide population-based cohort study, the use of SSRIs, SARIs, SNRIs, TCAs, NDRI, BZD, muscle relaxants, analgesic drugs, psychotherapies and exercise therapies were prescribed significantly more frequently in the CFS cohort than in the control group. Previous studies have reported these treatments to be effective at relieving the symptoms of CFS and useful for managing related comorbidities.

## Data Availability

The data underlying this study is from the National Health Insurance Research database (NHIRD). Interested researchers can obtain the data through formal application to the Ministry of Health and Welfare, Taiwan.
